# Acute Oral, Subacute, and Developmental Toxicity Profiling of Naphthalene 2-Yl, 2-Chloro, 5-Nitrobenzoate: Assessment Based on Stress Response, Toxicity, and Adverse Outcome Pathways

**DOI:** 10.3389/fphar.2021.810704

**Published:** 2022-01-20

**Authors:** Fareeha Anwar, Uzma Saleem, Atta ur rehman, Bashir Ahmad, Tariq Ismail, Muhammad Usman Mirza, Sarfraz Ahmad

**Affiliations:** ^1^ Riphah Institute of Pharmaceutical Sciences, Riphah International University, Lahore, Pakistan; ^2^ Riphah Institute of Pharmaceutical Sciences, Faculty of Pharmaceutical Sciences, Riphah International University, Islamabad, Pakistan; ^3^ Department of Pharmacology, Faculty of Pharmaceutical Sciences, Govt. College University, Faisalabad, Pakistan; ^4^ Department of Pharmacy, Forman Christian College, Lahore, Pakistan; ^5^ Department of Pharmacy, COMSATS Institute of Information Technology—Abbottabad Campus, Abottabad, Pakistan; ^6^ Department of Chemistry and Biochemistry, University of Windsor, Windsor, ON, Canada; ^7^ Drug Design and Development Research Group (DDDRG), Department of Chemistry, Faculty of Science, Universiti Malaya, Kuala Lumpur, Malaysia

**Keywords:** SF5, Nrf2, stress response pathway, acute oral toxicity, biochemical parameters, oxidative stress markers

## Abstract

The U.S. National Research Council (NRC) introduced new approaches to report toxicity studies. The NRC vision is to explore the toxicity pathways leading to the adverse effects in intact organisms by the exposure of the chemicals. This study examines the toxicity profiling of the naphthalene-2-yl 2-chloro-5-dinitrobenzoate (SF5) by adopting the vision of NRC that moves from traditional animal studies to the cellular pathways. Acute, subacute, and developmental toxicity studies were assayed according to the Organization for Economic Cooperation and Development (OECD) guidelines. The stress response pathway, toxicity pathway, and adverse effects outcome parameters were analyzed by using their standard protocols. The results showed that the acute toxicity study increases the liver enzyme levels. In a subacute toxicity study, alkaline phosphatase (ALP) levels were raised in both male and female animals. SF5 significantly increases the normal sperm count in the male animals corresponding to a decrease in the abnormality count. Developmental toxicity showed the normal skeletal and morphological parameters, except little hydrocephalus was observed in developmental toxicity. Doses of 20 mg/kg in males and 4 mg/kg in females showed decreased glutathione (GSH) levels in the kidney and liver. MDA levels were also increased in the kidney and liver. However, histopathological studies did not show any cellular change in these organs. No statistical difference was observed in histamine levels, testosterone, nuclear factor erythroid two-related factor-2 (Nrf2), and nuclear factor-kappa B (NF-κB), which showed no initiation of the stress response, toxicity, and adverse effect pathways. Immunomodulation was observed at low doses in subacute toxicity studies. It was concluded that SF5 did not produce abrupt and high-toxicity levels in organs and biochemical parameters. So, it is safe for further studies.

## 1 Introduction

The treatment of neurodegenerative diseases primarily includes control rather than cure due to potentially irreversible changes in the brain (Hussain et al., 2018). The anti-neurodegenerative drugs are usually used to alleviate symptoms or to retard disease progression. The prognosis of these diseases requires long-term, sometimes a lifetime, administration of the drugs. Hence, an anti-neurodegenerative drug must have a reliable safety profile, thus enabling it suitable for long-term administration. In an anti-neurodegenerative drug discovery campaign, apart from the desired efficacy, it is necessary to pay considerable attention to the side effects of the compounds under development.

It has been observed that a large number of drug candidates do not depict satisfactory preclinical toxicity profiles despite having promising *in vitro* and *in vivo* activities. In a drug discovery pipeline, preclinical toxicity analyses are of paramount significance for a drug to reach clinical practice. Based on the duration of the drug administration, preclinical toxicity studies are divided into acute toxicity, subacute toxicity, developmental toxicity, subchronic toxicity, genotoxicity, developmental toxicity, reproductive toxicity, cytotoxicity, and chronic toxicity. Recommended animal models and validated procedures are used for these studies. The conclusive goal of the toxicity studies is to correlate the animal responses toward humans (Gad, 2008).

A new era of science focuses on exploring the knowledge for the safety assessment of the new molecules at the molecular, genomic, cellular, and physiological levels. For this purpose, toxicity concepts were necessary for collecting the toxicity information in the biological system (Andersen et al., 2005). These pathways are the normal cellular response that becomes abnormal due to the exposure to the chemicals and produces adverse effects. Oxidative stress pathways and nuclear receptor-mediated pathways are the best examples of toxicity pathways (Krewski et al., 2020). These pathways identify the mechanistic approach of the toxicity potential of the chemical that causes the toxicity in animal studies, and these studies were correlated with the human relevance (Boobis et al., 2008). Nrf2 and NF-κB pathways play a pivotal role in regulating redox homeostasis, the primary response against oxidative stress, which is why these pathways play an essential role in toxicological studies. These pathways regulated the transcription of various genes, such as HO-1, NADPH quinone dehydrogenase-1 (NQO1), thioredoxin (TRX), glutathione, and superoxide dismutase (Mohsin Alvi et al., 2020). The lack of these pathways depleted the production of endogenous antioxidant enzymes such as glutathione and superoxide dismutase. This depletion leads to cell death even at the ordinary concentration of the reactive oxidant (Enomoto et al., 2001; Jennings, 2013).

**FIGURE 1:**
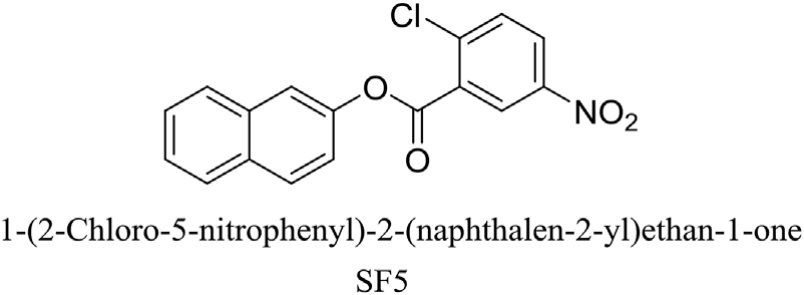
Chemical structure of SF5.

In our recently published work, an extensive *in silico* approach was used to identify cost-effective AChE inhibitors for treating Alzheimer’s disease. A series of potent naphthalene-based AChE inhibitors were identified, synthesized, and evaluated for their *in vitro* and *in vivo* potentials against Alzheimer’s disease, in which one of these inhibitors is SF5 (Anwar et al., 2020). Our previous study is the pilot study, and before the detailed *in vivo* study against Alzheimer’s disease, a brief toxicity profiling is necessary. For this reason, this study was designed to explore the single dose, repeated dose, and reproductive toxicity potential of one of these compounds naphthalene-2-yl 2-chloro-5-dinitrobenzoate (SF5) (Figure 1) and its effect on oxidative stress and toxicity pathways.

## 2 Materials and Methods

### 2.1 Drugs and Chemicals

Pyrogallol solution and Alcian blue were purchased from Oxford Labs (India). Elman’s reagent and Alizarin red S were purchased from Omicron Sciences Limited (United Kingdom). Follin’s reagent, carboxymethylcellulose, picric acid, EDTA, N-1-naphthyl ethylene amine dihydrochloride, sulfanilamide, thiobarbituric acid, sodium-potassium tartrate, DTNB, and Griess reagent were purchased from Sigma-Aldrich, United States. Histamine and testosterone were purchased from ThermoFisher Scientific, United States. All chemicals and solvents used in the study were of analytical or pharmaceutical grade.

### 2.2 Experimental Animals

Adult Wistar rats, 2–3 months old, weighing 100–280 g, were used in the study. The animals were housed in the animal house of Riphah Institute of Pharmaceutical Sciences, Riphah International University’s Lahore campus. The experimental animals were provided with the standard environmental conditions of temperature 22 ± 2°C, 40–50% humidity, 12/12 h light/dark cycles, and free access to food and water. All the experimental protocols of toxicity studies (acute oral, subacute oral, and developmental toxicity) were approved by the Ethical Committee of Riphah Institute of Pharmaceutical Sciences, Lahore Campus, with the voucher number of REC/RIPS-LHR/035 for further considerations under the rules and regulations of the National Institute of Health (NIH) Guide for the Care and Use of Laboratory Animals.

### 2.3 Acute Oral Toxicity

An acute oral toxicity study was performed, followed by the Organization for Economic Cooperation and Development (OECD) guidelines 425 of chemicals. Ten female rats (100–250 g weight) were randomly divided into two groups (*n* = 5). Group 1 served as the control group and received 1 ml/kg 0.5% carboxymethylcellulose (CMC), while group II was treated with a dose of 2,000 mg/kg SF5. First, the animals were orally treated with a single SF5 dose of 2,000 mg/kg. The animal was critically observed for 24 h. If no morbidity and mortality were observed, then the other four animals were given their respective dose. Toxicity was observed for 14 days in total. Gross observations made to detect the toxicity were general behavior, skin, fur, and eye changes, secretions from the mucous membrane, respiratory and autonomic or CNS disturbances, morbidity, and mortality. After 14 days, female rats were anesthetized using isoflurane (2–3%) diluted with oxygen, and blood was collected by cardiac puncture (Abozeid et al., 2020).

### 2.4 Subacute Toxicity

Healthy male and female rats (*n* = 10) weighing 100–200 g were used in this study. Each group consisted of an equal number of male and female rats. Group 1 received 1 ml/kg CMC and was designated as control; group II–V received SF5 at 5, 10, 20, and 40 mg/kg dose levels, respectively. Treatments were given once daily for 28 days through the oral route. Any change in body weight and physical appearance was observed throughout the study period (Xiao et al., 2019).

### 2.5 Developmental Toxicity

The highest dose of SF5 (40 mg/kg) used in the subacute toxicity study was selected for the teratogenic studies. An OECD 414 guideline for developmental toxicity was used to design the protocol. Twenty female rats (200–280 g) were divided into two groups (*n* = 10): a control group and a treatment group (SF5, 40 mg/kg orally). Three female rats were housed with one male and observed for a vaginal plug, i.e., the start of gestation (day zero). Treatments were continued from gestational day 5–15 *via* the oral route. The C-section was performed on the 19th day of gestation, and fetuses were removed carefully (Saeed et al., 2020). Fetuses and placenta were weighed, and any deformity in fetuses was recorded (Buschmann, 2013; Saeed et al., 2020).

### 2.6 Staining of the Fetal Skeleton

Fetuses were soaked into 4% normal saline solution overnight to remove skin, muscles, and organs from the skeleton (Menegola et al., 2001). After removing the muscular mass, the skeleton was stained by acidic staining (Alizarin red, pH 2.8) for up to 24 h. After staining, moisture was evaporated by soaking the skeleton into the absolute alcohol. After dehydration, the specimen was soaked into the basic stain (Alcian blue) for up to 24–30 h. The stained specimen was then placed into the clearing solution (1:1, 70% ethanol:glycerin) for 6–8 h. The samples were analyzed under a dissecting microscope for analyzing the bone ossification, spina bifida, ribs and limbs deformities, and cleft palate (Ehlers et al., 1996; Menegola et al., 2001).

### 2.7 Soft Tissue Examination

Wilson’s technique was used for the rapid and gross examination of soft tissues. Fetuses were soaked for 8–10 days in the Bouin’s solution (saturated solution of picric acid). After 8–10 days of soaking, the fetuses were soaked in distilled water to remove picric acid. The skin was removed, and the tissues were observed separately. Different transverse and longitudinal cuts were made for the head analysis. Organs were examined for any megaly and visual abnormality (Barrow and Taylor, 1969).

### 2.8 Sperm Analysis

The diffusion method was used to collect sperm samples in the subacute toxicity study. Orchidectomy was performed by the castration method. The testis at the pre-scrotal region was incised, and the testicles were oozed out. The cauda epididymis was poured into the Petri dish containing phosphate buffer at pH 7.4. Sperm suspension was made by swirling the Petri plate, and the suspension was analyzed for sperm count and morphological features (Saleem et al., 2020).

### 2.9 Hematological Analysis

After each toxicity study, animals were given anesthesia using the 2–3% isoflurane diluted with oxygen. Blood was collected through cardiac puncture. Hematological parameters (RBCs, WBCs, platelets, hemoglobin, MCH, MCV, and % leukocyte) were determined (Yang et al., 2019).

### 2.10 Biochemical Analysis

Blood (2–3 ml) was poured into the EDTA tube and centrifuged at 4,000 rpm for 15 min. The resulting plasma was stored for biochemical analysis. Biochemical parameters, AST, ALT, ALP, bilirubin, urea, creatinine, HDL, LDL, VLDL, and total cholesterol, were measured using kits through a chemistry analyzer (Merck, United States) (Rodríguez-Lara et al., 2019).

The blood sample was placed in an EDTA-free tube and centrifuged at 4,000 rpm for 15 min to collect serum. The serum was separated, and TSH, T3, and T4 levels were measured using ELISA kits (Biocompare, San Francisco, United States).

### 2.11 Oxidative Stress Biomarkers

After the blood collection, animals were sacrificed by the cervical dislocation method. The selected organs (brain, kidney, heart, liver, ovary, testis, and spleen) were removed and weighed. The tissue homogenates of these organs were prepared in the phosphate buffer (0.1 M, pH 7.4, 4°C) with a ratio of 1:10. The homogenate was centrifuged at 6,000 rpm and 4°C for 10 min. The supernatant was collected to estimate SOD, CAT, GSH, and MDA levels (Majeed et al., 2020).

### 2.12 Superoxide Dismutase

The tissue homogenate (0.1 ml), pyrogallol solution (0.1 ml), and potassium phosphate buffer (2.8 ml, pH 7.4, 0.1 M) were mixed thoroughly in a test tube. Absorbance was measured at 325 nm (Parambi et al., 2020). The levels of SOD were calculated by using the following regression line of the standard:

Y=0.0095X+0.1939.

### 2.13 Catalase

Phosphate buffer (1.95 ml, pH 7, 50 mM) and tissue homogenate (50 µL) were mixed in H_2_O_2_ (1 ml, 30 mM) solution. The mixture was thoroughly mixed using a vortex, and absorbance was measured at 240 nm (Parambi et al., 2020). The following formula was used for CAT levels measurements:

$$CAT\;levels=\frac{OD}{E}\times vol.\;of\;sample\times mg\;of\;protein,$$

where OD is the change in absorbance, and E is the extinction coefficient of H_2_O_2_ (0.071 mmol/cm). Total protein was estimated using the Lowery method (Nazir et al., 2021).

### 2.14 Total Glutathione

Tissue homogenate (1 ml), trichloroacetic acid (10%, 1 ml), 4 ml phosphate buffer (0.1 M, pH 8), and 0.5 ml of DTNB (0.1 mM) solution were mixed. After precipitation, the supernatant was removed, and absorbance was measured at 412 nm (Hira et al., 2020). The GSH levels were measured by using the following formula:

$$GSH=Y-\frac{0.00314}{0.034}\times \frac{DF}{BT}\times VU,$$

where DF = dilution factor, BT = tissue homogenate, VU = volume used, and Y = absorbance at 412 nm.

### 2.15 Malondialdehyde

Thiobarbituric acid (3 ml, 4.0 mM) was added in tissue homogenate (1 ml), mixed well, and incubated at room temperature for 15 min. The reaction mixture was heated at 80°C for 15 min and cooled on an ice bath. The supernatant was separated, and its absorbance was measured at 532 nm. The levels of MDA were analyzed by using the following formula (Saeed et al., 2020):

Conc.of MDA=Abs 532×100×Vt/1.56×105×Wt×Vu,

where V_T_ = total volume of mixture (i.e., 4 ml), W_T_ = weight of dissected brain, and V_U_ = aliquot volume.

### 2.16 Histamine, Nrf2, NF-κB, and Testosterone

In the subacute toxicity study, blood serum of both male and female rats was used to estimate histamine, Nrf2, NF-κB, and testosterone parameter levels. Each targeted parameter was combined with the HRP-labeled antibody to make an antigen–antibody complex. The complex was mixed with TBM solution, and the reaction was stopped by adding the stop solution. Absorbance was measured at 450 nm using an ELISA reader. The levels of histamine, Nrf2, NF-κB, and testosterone were calculated by using their specific standard regression lines (DeMattos et al., 2002; Ali et al., 2020).

### 2.17 Histopathological Studies

At the end of acute oral toxicity, subacute toxicity, and developmental toxicity studies, selected organs were fixed in 4% formaldehyde solution, embedded in paraffin wax, and sliced. Sliced sections were fixed on slides and stained with HE staining. The tissue sections were observed under a microscope (100X) for analyzing any change in the cells (Justin Thenmozhi et al., 2015).

### 2.18 Statistical Analysis

All the data were expressed as mean ± SEM. GraphPad Prism 8.4 software was used for the interpretation of the experimental data. One-way ANOVA or two-way ANOVA was used to analyze the data, followed by the Tukey comparison and Bonferroni *post hoc* test. *p < 0.05 was considered as the level of significance. **p < 0.01 and ***p < 0.001 were labeled as moderate and highly significant levels, respectively.

## 3 Results

### 3.1 Acute Toxicity

#### 3.1.1 Behavioral and Physical Changes

No toxicity symptoms in behavioral parameters such as skin, fur, and eye changes, secretions from the mucous membrane, respiratory and autonomic or CNS disturbances, morbidity, and mortality were observed. No morbidity or mortality was observed at a dose of 2,000 mg/kg of SF5, illustrating its *LD*_*50*_ higher than 2,000 mg/kg.

#### 3.1.2 Body and Organ Weights

**FIGURE 2:**
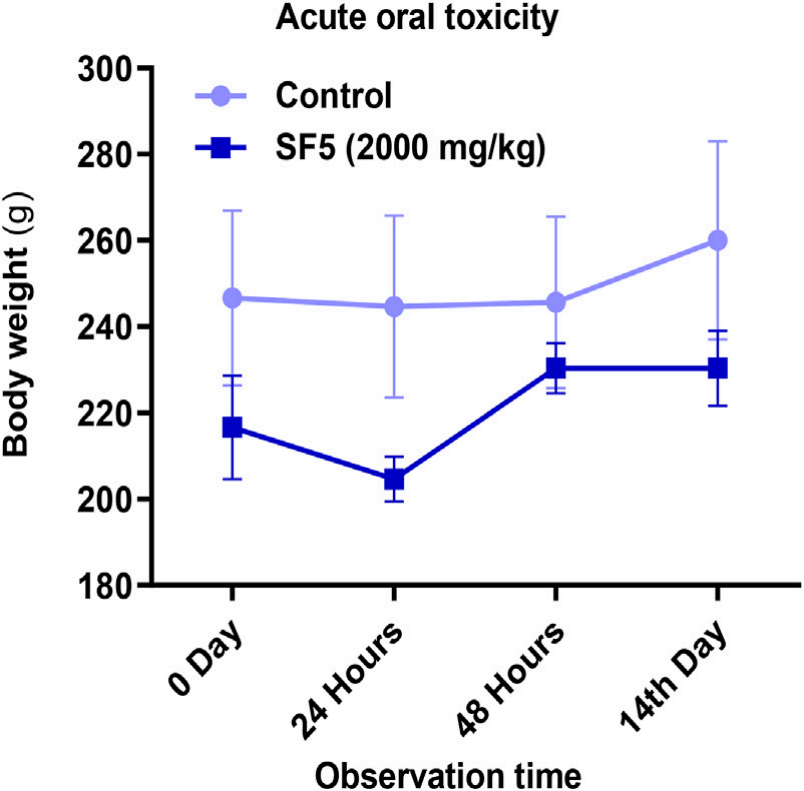
Effect of SF5 on body weights of rats in different toxicity studies.

The body weights of the animals during the course of acute toxicity were recorded at start, 24 h, 48 h, and 14th day (Figure 2). No considerable changes in the body weights were observed compared to those of the control.

After the completion of 14-days trial, the animals were dissected and the organ weights were recorded (Table 1). The weights of the brain, lungs, and liver were increased significantly, while the remaining organs were not considerably different compared to those of the control.

**TABLE 1:** Effect of different doses of SF5 treatment on the organ weights of selected organs in acute toxicity studies.

Organ	Acute toxicity	
	Control	2,000 mg/kg
Brain	1.5 ± 0.2	1.66 ± 0.2*
Heart	0.3 ± 0.1	0.44 ± 0.1
Spleen	0.2 ± 0.1	0.3 ± 0.8
Kidney	1.2 ± 0.1	1.7 ± 0.1
Lungs	0.7 ± 0.3	0.92 ± 0.3*
Liver	6.9 ± 0.4	7.4 ± 0.5*
Stomach	0.9 ± 0.8	1.1 ± 1.2
Ovaries	0.2 ± 1.5	0.1 ± 0.9

Data are presented as mean ± SEM, *n* = 5. *p < 0.05 is a level of increase in comparison to the control.

#### 3.1.3 Oxidative Stress

Different levels of oxidative stress were observed in the selected organs in the acute toxicity study (Table 2). Among the selected organs, the liver was the most affected one with the disturbed value of all the four tested parameters. SOD was slightly decreased and CAT was considerably reduced, while GSH and MDA were significantly increased compared to those of the normal control. The kidney presented a significant decrease in CAT and an increase in MDA. In the stomach, the only disturbed parameter was the decreased value of CAT. The spleen was the second most affected organ, showing decreased SOD and CAT and increased GSH. The most significant decrease in SOD was observed in the brain, where GSH was also significantly increased.

**TABLE 2:** Effect of SF5 treatment on oxidative stress markers in the acute toxicity study.

Organ	Treatment group	SOD (µg/mg of tissue protein)	GSH (µg/mg of tissue protein)	CAT (mmole/min/mg of protein)	MDA (µmole/mg of tissue protein)
Liver	Normal	9.97 ± 0.005	0.147 ± 0.014	2.03 ± 0.79	0.04 ± 0.05
	SF5	7.85 ± 0.75^	1.949 ± 1.1*	0.533 ± 1.2^	0.208 ± 0.34*
Kidney	Normal	28.57 ± 0.46	2.050 ± 0.011	4.53 ± 0.54	2.050 ± 0.011
	SF5	26.01 ± 1.0	1.785 ± 0.87	1.11 ± 0.32^	4.6 ± 0.32*
Stomach	Normal	1.1 ± 0.2	1.1 ± 0.2	1.5 ± 1.3	0.1 ± 0.24
	SF5	0.92 ± 1.2	0.1 ± 1.2	0.349 ± 2.1^	0.277 ± 0.85
Spleen	Normal	42.54 ± 1.2	0.12 ± 0.3	1.2 ± 2.1	0.2 ± 1.3
	SF5	34.85 ± 2.3^	1.814 ± 1.03*	0.803 ± 3.2^	0.20 ± 1.2
Brain	Normal	28.62 ± 0.51	0.133 ± 0.011	0.654 ± 2.63	1.558 ± 0.011
	SF5	10.65 ± 1.6^	1.090 ± 0.32*	0.519 ± 0.89	0.767 ± 0.56

Data are presented as mean ± SEM, *n* = 3. *p < 0.05 is an increase in the levels of significance, while ^*p* < 0.05 is a level of decrease in comparison to the control.

#### 3.1.4 Biochemical Marker

The biochemical markers were selected to investigate kidney function, liver function, and lipid profile (Table 3). In terms of kidney function, the total protein content was 1.7 times higher than that of the control. Uric acid, urea, and creatinine were significantly decreased compared to those of the control. In liver function parameters, all the tested analytes were increased with around two-time increase in AST and three-time increase in ALP and ALT. The lipid profile was relatively less disturbed than the liver and kidney function. Cholesterol, HDL, and LDL were decreased, while triglycerides were slightly increased.

**TABLE 3:** Biochemical marker analysis after SF5 treatment in the acute oral toxicity study.

Biochemical marker	Unit	Control	SF5 (2,000 mg/kg)
Uric acid	mg/dL	5.0 ± 1.2	1.25 ± 1.02
Urea	mg/dL	30.2 ± 0.12	6.8 ± 1.08
Protein	g/dL	7.65 ± 1.2	13.30 ± 0.65*
Creatinine	mg/dL	1.12 ± 2.1	0.71 ± 0.87
Bilirubin	mg/dL	1.2 ± 1.42	1.41 ± 1.05
ALP	U/L	185 ± 1.97	557 ± 2.03***
ALT	U/L	42 ± 0.34	120 ± 0.65***
AST	U/L	65 ± 1.45	116 ± 2.3***
Cholesterol	mg/dL	65.12 ± 2.1	44 ± 3.0
HDL	mg/dL	20.3 ± 1.23	10.5 ± 0.65
Triglyceride	mg/dL	56.84 ± 1.2	62.1 ± 0.98
LDL	—	33.32 ± 0.32	21.6 ± 0.32

Data are presented as mean ± SEM, *n* = 5. *p < 0.05, ***p < 0.001 compared to the control.

#### 3.1.5 Histopathology

**FIGURE 3:**
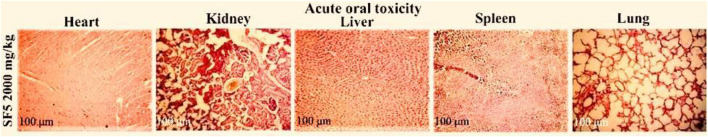
Histopathology of the selected organs in acute oral toxicity (2,000 mg/kg).

The representative slides of the histopathological analysis of the selected organs are presented in Figure 3. All the organs showed normal histology except the kidney. Necrosis and inflammation were observed in the kidney, while the glomerulus and Bowman’s capsule were not in their intact state.

### 3.2 Subacute Toxicity

#### 3.2.1 Body and Organ Weights

**FIGURE 4:**
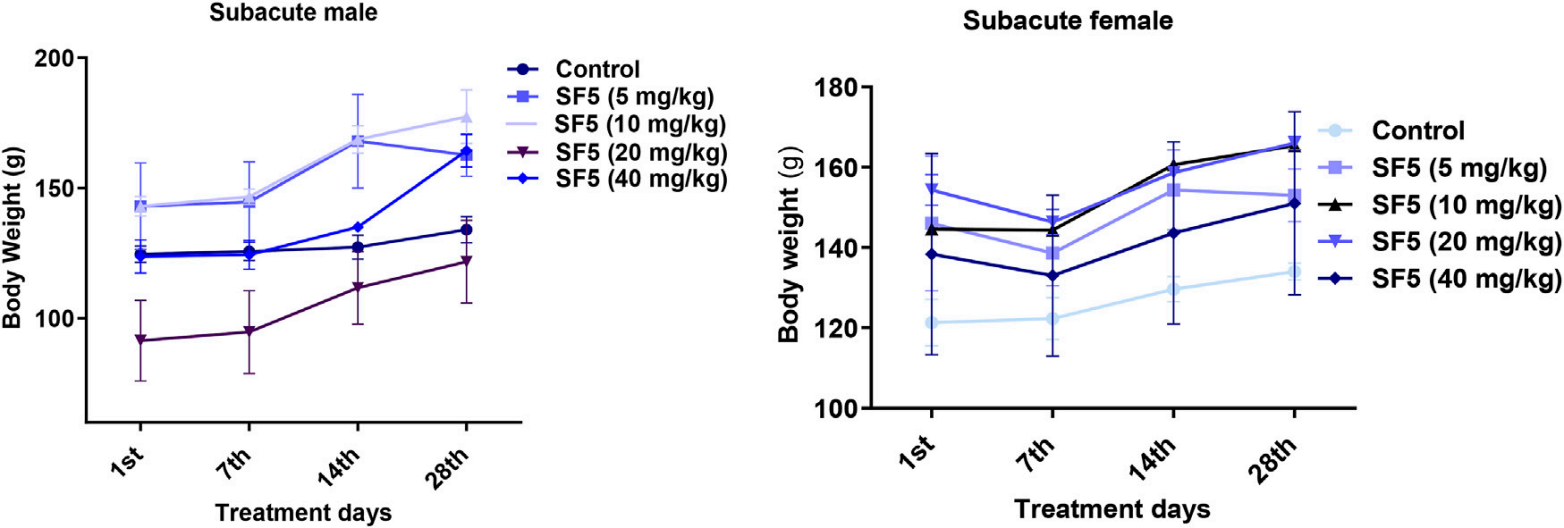
Effect of SF5 on body weights of rats in the subacute toxicity study.

The change in body weights of the male and female rats during the subacute toxicity trial is presented in Figure 4. The body weights stayed well within the limits during the 28-days trial.

On repeated-dose administration and sex differentiation effect studies (subacute toxicity), only males at 10 and 40 mg/kg doses showed increased kidney weight. In contrast, the weights of all other tissues were in the normal range with no change in color and weight (Table 4).

**TABLE 4:** Effect of different doses of SF5 treatment on the organ weights of selected organs in the subacute toxicity study.

Organ	Weight of the organs (g) in subacute toxicity									
	Male					Female				
	Control	5 mg/kg	10 mg/kg	20 mg/kg	40 mg/kg	Control	5 mg/kg	10 mg/kg	20 mg/kg	40 mg/kg
Brain	1.3 ± 0.3	1.3 ± 0.1	1.4 ± 0.2	1.1 ± 0.6	1.4 ± 0.6	1.5 ± 0.4	1.6 ± 0.6	1.5 ± 0.2	1.5 ± 0.3	1.4 ± 0.1
Heart	0.5 ± 0.2	0.6 ± 0.2	0.5 ± 0.3	0.2 ± 0.8	0.6 ± 0.5	0.6 ± 0.3	0.5 ± 0.3	0.6 ± 0.1	0.6 ± 0.2	0.6 ± 0.3
Spleen	0.3 ± 0.4	0.5 ± 0.3	0.4 ± 0.4	0.2 ± 03	0.4 ± 0.3	0.4 ± 0.1	0.5 ± 0.3	0.4 ± 0.2	0.4 ± 0.3	0.3 ± 0.5
Kidney	1.2 ± 0.3	1.2 ± 0.2	1.5 ± 0.8*	1.1 ± 0.5	1.4 ± 0.1*	1.1 ± 0.5	1.1 ± 0.2	1.2 ± 0.1	1.1 ± 0.5	1.3 ± 0.3
Lungs	0.8 ± 0.5	0.8 ± 1.3	0.8 ± 0.6	0.5 ± 0.7	0.8 ± 0.6	0.9 ± 0.2	0.7 ± 0.1	0.7 ± 0.2	0.6 ± 0.3	0.8 ± 0.8
Liver	5.8 ± 0.5	6.9 ± 0.1	6.3 ± 1.2	5.9 ± 0.3	6.6 ± 0.2	6.1 ± 0.4	6.6 ± 0.3	6.2 ± 0.3	6.9 ± 0.6	6.7 ± 0.5
Stomach	1.1 ± 0.5	1.2 ± 0.0	1.6 ± 0.9	1.0 ± 0.8	1.2 ± 0.3	1.2 ± 0.3	1.2 ± 0.5	1.3 ± 0.5	1.5 ± 0.7	1.6 ± 0.8
Ovaries	—	—	—	—	—	0.1 ± 0.1	0.1 ± 1.2	0.1 ± 0.1	0.1 ± 0.1	0.2 ± 0.9
Testis	2.6 ± 0.4	2.2 ± 0.3	2.2 ± 0.5	2.1 ± 0.7	2.1 ± 0.1	—	—	—	—	—

Data are presented as mean ± SEM, *n* = 10. *p < 0.05 is a level of increase in comparison to the control.

#### 3.2.2 Hematological Parameters

At the end of the subacute toxicity study, the blood samples were collected to determine the change in the hematological parameter due to the administration of SF5. The results are listed in Table 5. Increased white blood cell and differential leukocyte counts were observed in SF5-treated female animals at 10 mg/kg dose levels. The same scenario was observed in male animals treated with SF5. All doses significantly increased the levels of WBCs and their differential count. In contrast, SF5 at 5, 10, and 20 mg/kg doses substantially decreases the platelet count, while SF5 at 40 mg/kg substantially increases the platelet count in females.

**TABLE 5:** Hematological parameters measured in male and female rats during subacute toxicity study at different doses of SF5.

Treatment group	WBCs (×10^3^/µL)	RBCs (×10^6^/µL)	Platelets (×10^3^/µL)	HB (g/dl)	LYM (×10^3^/µL)	MID (×10^3^/µL)	GRA (×10^3^/µL)	MCH (pg)	MCHC (g/dl)	MCV (fL)	HCT (%)
Female											
Control	3.7 ± 0.12	5.95 ± 0.32	817 ± 0.14	12.0 ± 1.2	3.1 ± 1.4	0.4 ± 1.3	0.1 ± 0.02	20.30 ± 0.15	36.5 ± 0.96	55.4 ± 1.2	33.0 ± 3.2
SF5 (5)	4.3 ± 1.2	4.74 ± 3.2	316 ± 0.21^	9.7 ± 0.014^	3.3 ± 1.08	0.4 ± 0.5	0.6 ± 0.32*	20.50 ± 0.23	34.9 ± 0.85	58.8 ± 0.2	27.9 ± 1.3
SF5 (10)	7.0 ± 0.12*	6.75 ± 0.65*	502 ± 0.52^	12.8 ± 0.32	2.3 ± 2.1	3.5 ± 1.3*	1.1 ± 2.1*	18.90 ± 0.87	34.1 ± 0.02	55.5 ± 1.0	37.5 ± 2.1
SF5 (20)	3.1 ± 2.1	6.45 ± 1.3*	553 ± 0.03^	12.9 ± 0.5	2.5 ± 3.2	0.5 ± 1.9	0.1 ± 0.89	20.10 ± 0.79	35.5 ± 2.1	56.6 ± 1.3	36.3 ± 1.0
SF5 (40)	1.5 ± 0.53^	7.75 ± 1.3*	854 ± 3.1	14.0 ± 0.35	1.1 ± 1.5^	0.1 ± 0.3^	0.2 ± 0.96	18.0 ± 1.3	34.6 ± 2.3	52.0 ± 1.0	40.3 ± 1.0*
Male											
Control	4.2 ± 0.2	6.25 ± 0.3	627 ± 0.4	11.5 ± 1.2	3.1 ± 1.4	0.4 ± 1.3	0.1 ± 0.02	18.90 ± 0.15	38.3 ± 0.96	54.4 ± 1.2	33.0 ± 3.2
SF5 (5)	7.9 ± 1.2*	6.53 ± 3.2	714 ± 0.21	13.8 ± 0.014	6.7 ± 1.08*	0.8 ± 0.5	0.4 ± 0.32	21.10 ± 0.23	36.0 ± 0.85	58.7 ± 0.2	38.3 ± 1.3
SF5 (10)	7.4 ± 0.12*	6.54 ± 0.65	541 ± 0.52^	13.2 ± 0.32	5.7 ± 2.1*	1.4 ± 1.3*	0.3 ± 2.1	20.20 ± 0.87	35.8 ± 0.02	56.4 ± 1.0	36.9 ± 2.1
SF5 (20)	5.6 ± 2.1*	5.48 ± 1.3	966 ± 0.03*	11.5 ± 0.5	4.5 ± 3.2	0.9 ± 1.9	0.3 ± 0.89	20.90 ± 0.79	34.6 ± 2.1	60.4 ± 1.3	33.1 ± 1.0
SF5 (40)	8.1 ± 3.2*	6.76 ± 1.2	717 ± 0.2	13.1 ± 0.3	6.1 ± 1.2*	1.6 ± 0.5*	0.4 ± 1.3	19.40 ± 2.3	35.3 ± 2.5	55.0 ± 1.2	37.2 ± 3.0

Data are represented as mean ± SEM, *n* = 5. *p < 0.05 is the level of increase and ^*p* < 0.05 is the level of decrease compared to the control group.

#### 3.2.3 Biochemical Markers

**FIGURE 5:**
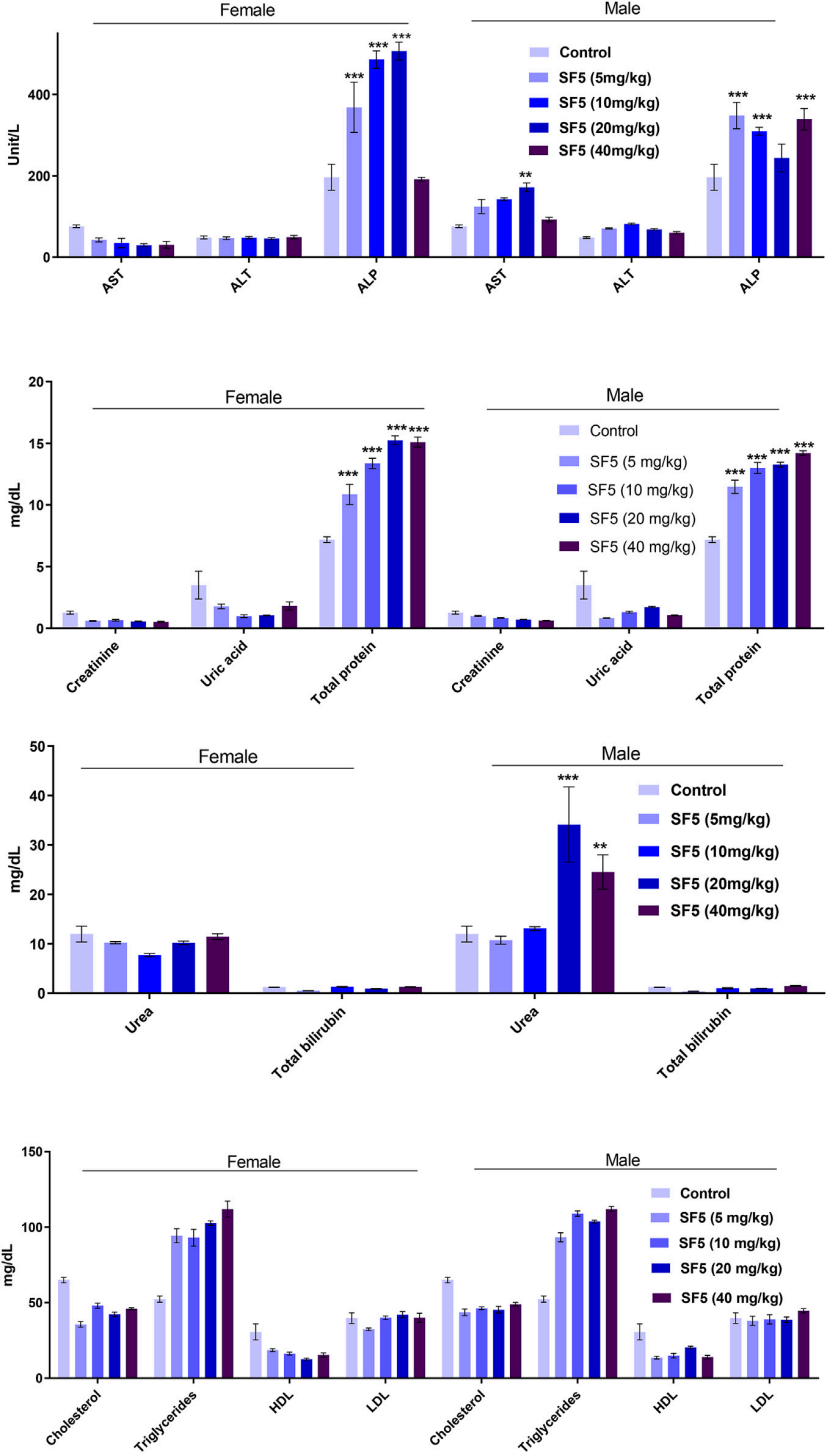
Effect of SF5 treatment on liver function, kidney function, and lipid profile in both males and females at different dose levels. **p < 0.01 and ***p < 0.001 given in comparison to the control.

Biochemical markers estimation is necessary to determine the toxicity levels in the major organs (liver and kidney) (Figure 5). The alkaline phosphatase (ALP) level was significantly (*p* < 0.001) increased in both male and female animals. A dose-dependent increase was observed as the dose was increased from 5 mg/kg to 40 mg/kg, and the value of ALP was raised accordingly. The level of protein was also raised in a dose-dependent manner in both male and female rats. No change in the lipid profile was observed in all treated animals. The urea level was little bit increased in males at 20 and 40 mg/kg doses.

**FIGURE 6:**
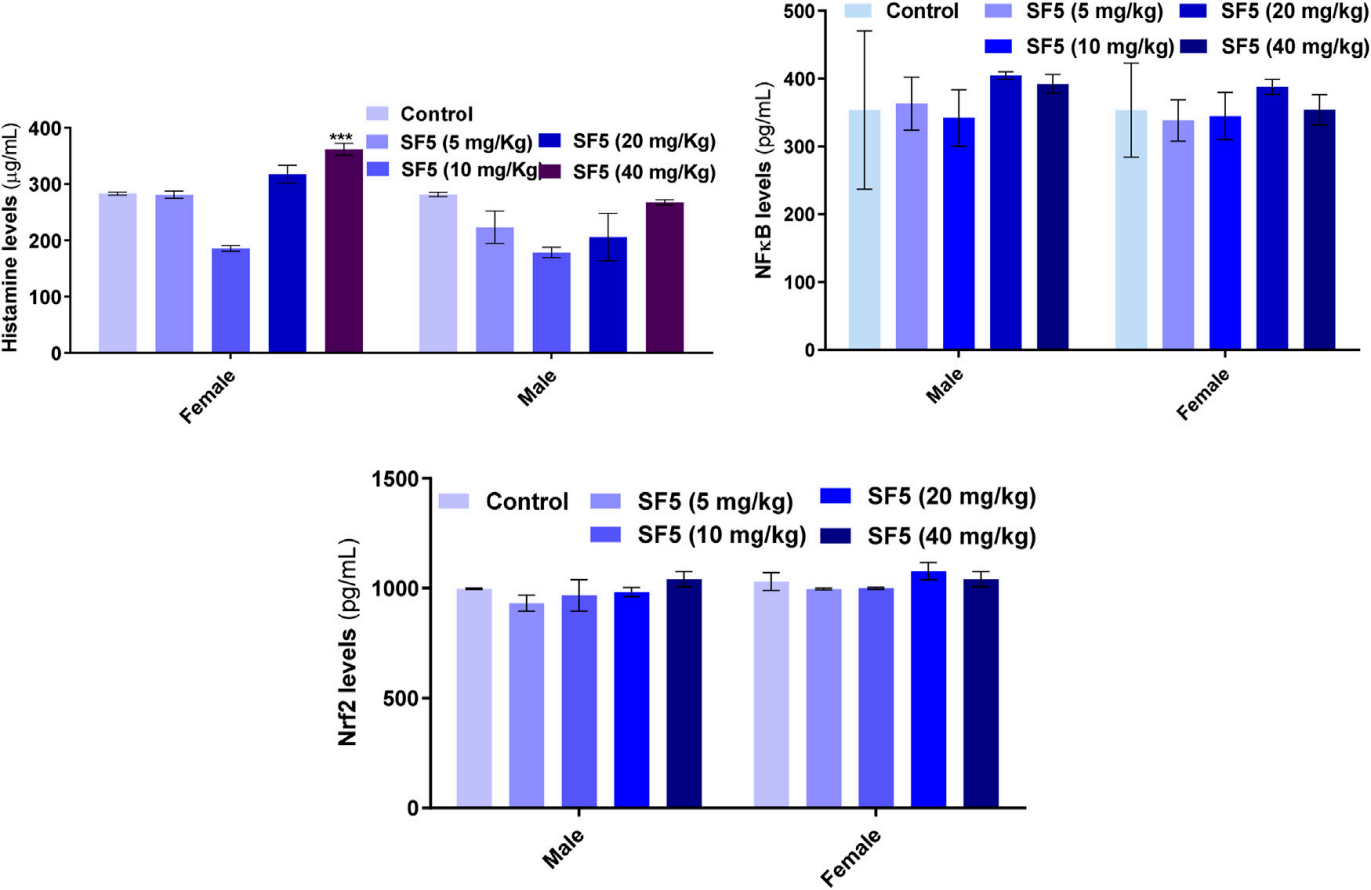
Effect of repeated-dose administration of a different dose of SF5 on histamine, NF-κB, and Nrf2 levels in the serum of the treated animals in subacute toxicity studies. Data are presented as mean ± SEM, *n* = 3, ***p < 0.01 compared to the control.

Figure 6 showed that the histamine level was increased significantly in females at the highest dose (40 mg/kg). In contrast, all other doses did not lead to any change in the histamine level in both male and female animals. No toxic effects were observed in Nfr2 and NF-κB levels.

#### 3.2.4 Oxidative Stress

**FIGURE 7:**
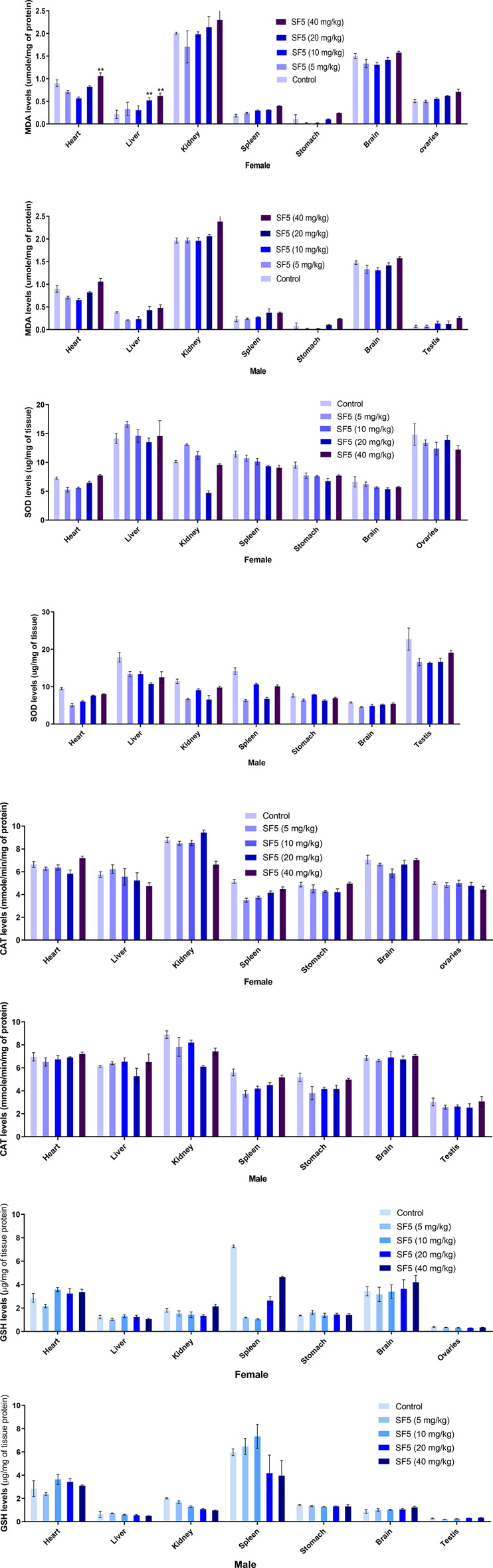
Estimation of oxidative stress markers in the subacute toxicity study in male and female rats. Data are presented as mean ± SEM, *n* = 3. *p < 0.05 is the increase in the level of significance while ^*p* < 0.05 is the level of decrease in comparison to the control.

Different oxidative stress markers (SOD, CAT, GSH, and MDA) were estimated at the end of the subacute toxicity study to determine the toxicity at the cellular level Figure 7. The levels of SOD were decreased significantly in the liver, kidney, and spleen tissues of male rats, while no effect was observed in females. A similar pattern was seen in the levels of CAT as decreased in the kidney and liver of male rats except for the spleen. No effect was observed in the levels of GSH at any organ. However, an increase in MDA levels was analyzed in the higher doses of the female rats. All observations were made in comparison to control.

#### 3.2.5 Histopathology

**FIGURE 8:**
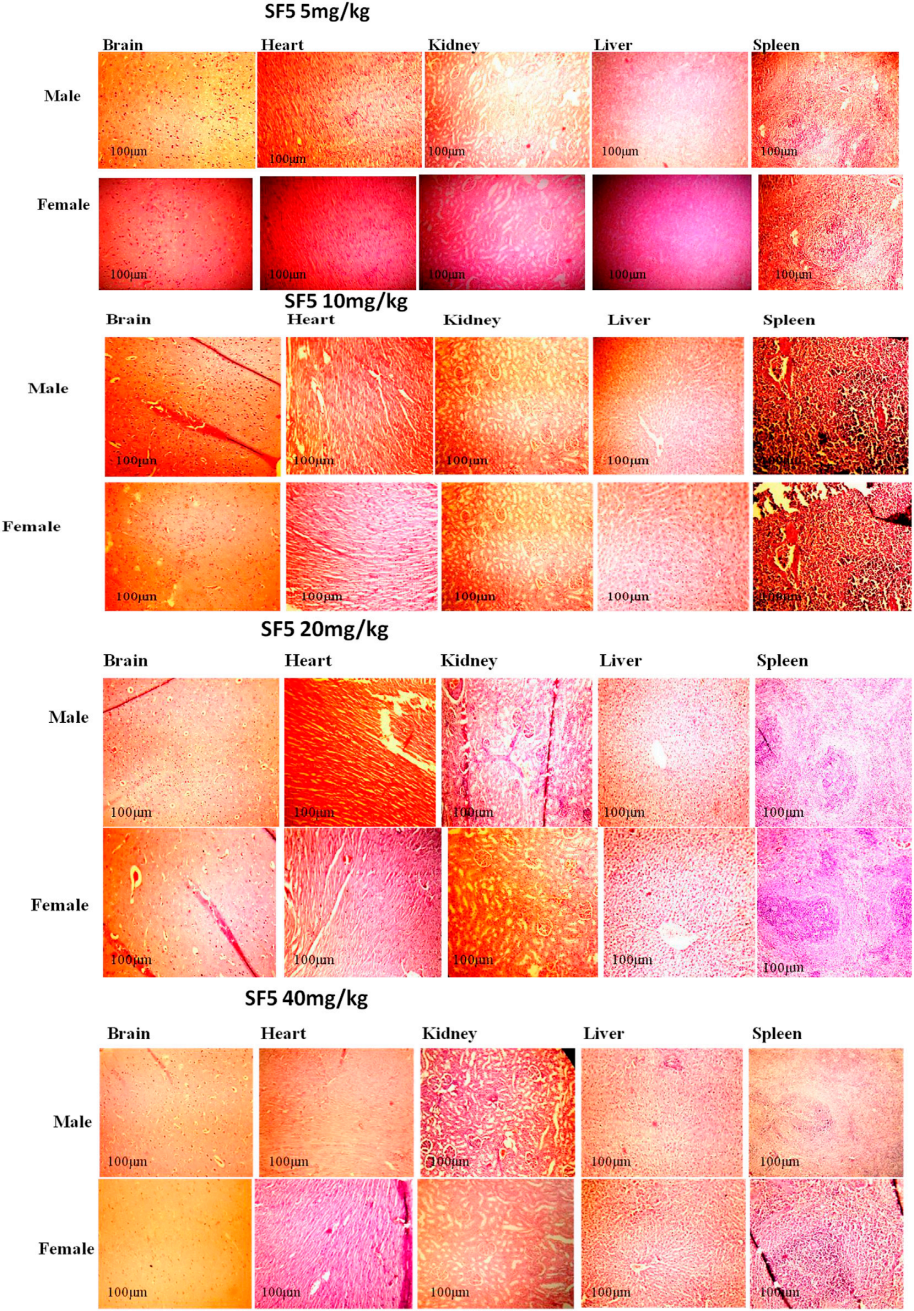
Histopathological analysis of different organs in subacute toxicity studies of SF5 at 5, 10, 20, and 40 mg/kg dose levels.

The selected organs (brain, heart, kidney, liver, and spleen) were analyzed for the change in the tissue architecture induced by the administration of different dose levels (5, 10, 20, and 40 mg/kg) of SF5. The representative slides with maximum distortion in tissue architecture, if any, are presented in Figure 8. All the selected organs showed normal histology. No sign of toxicity was observed in any organ of the treated animals.

### 3.3 Developmental Toxicity

#### 3.3.1 Body and Organ Weights

**FIGURE 9:**
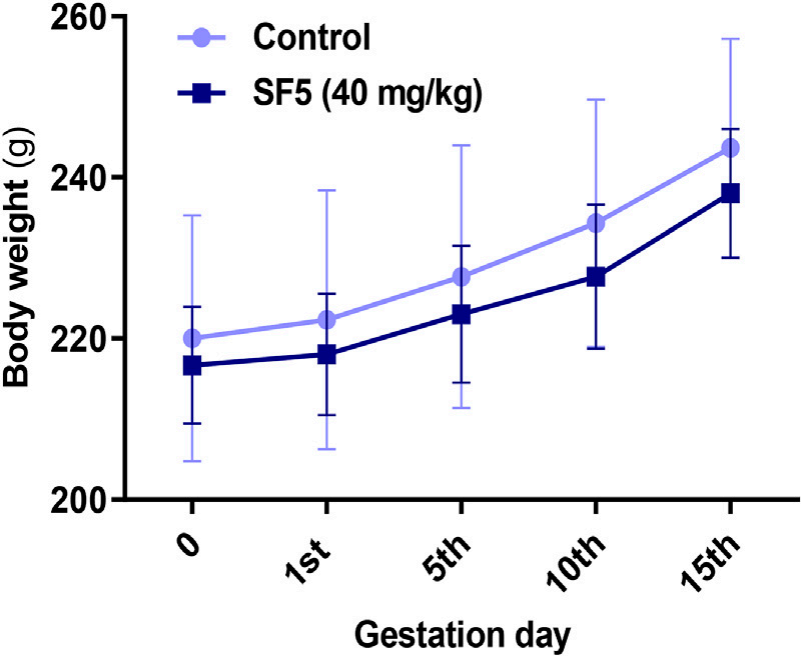
Effect of SF5 on body weights of rats in the developmental toxicity study.

The effect of SF5 on the body weights in the developmental toxicity study is given in Figure 9. The differences in body weights were statistically insignificant compared to those of the control.

At the end of the developmental toxicity study, the animals were dissected and weights of the selected organs were recorded Table 6. There was no significant difference in organ weights compared to those of the control group.

**TABLE 6:** Effect of SF5 treatment on the weights of selected organs in the developmental toxicity study.

Organ	Developmental toxicity	
	Control	40 mg/kg
Brain	1.4 ± 0.2	1.5 ± 0.12
Heart	0.62 ± 0.3	0.6 ± 0.32
Spleen	0.54 ± 0.1	0.53 ± 0.40
Kidney	1.2 ± 0.1	1.23 ± 0.13
Lungs	0.75 ± 0.2	0.16 ± 0.52
Liver	6.24 ± 1.2	6.7 ± 1.3
Stomach	1.2 ± 0.2	0.90 ± 0.62
Ovaries	0.32 ± 0.4	0.23 ± 0.3

Data are presented as mean ± SEM, *n* = 10.

#### 3.3.2 Effect on Fetus

**FIGURE 10:**
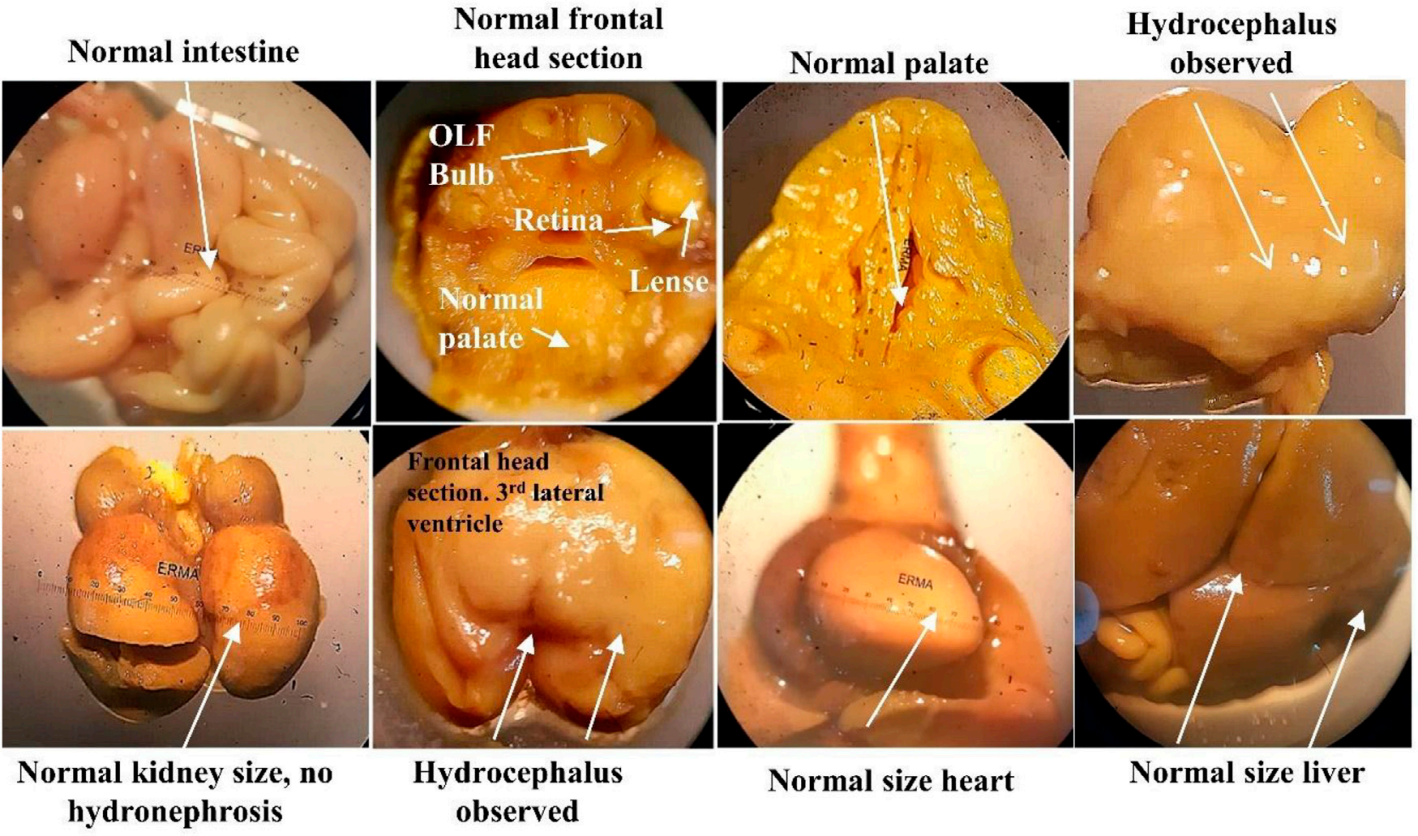
Soft tissue examination of fetus through Bouin fixation in the developmental toxicity study.

**FIGURE 11:**
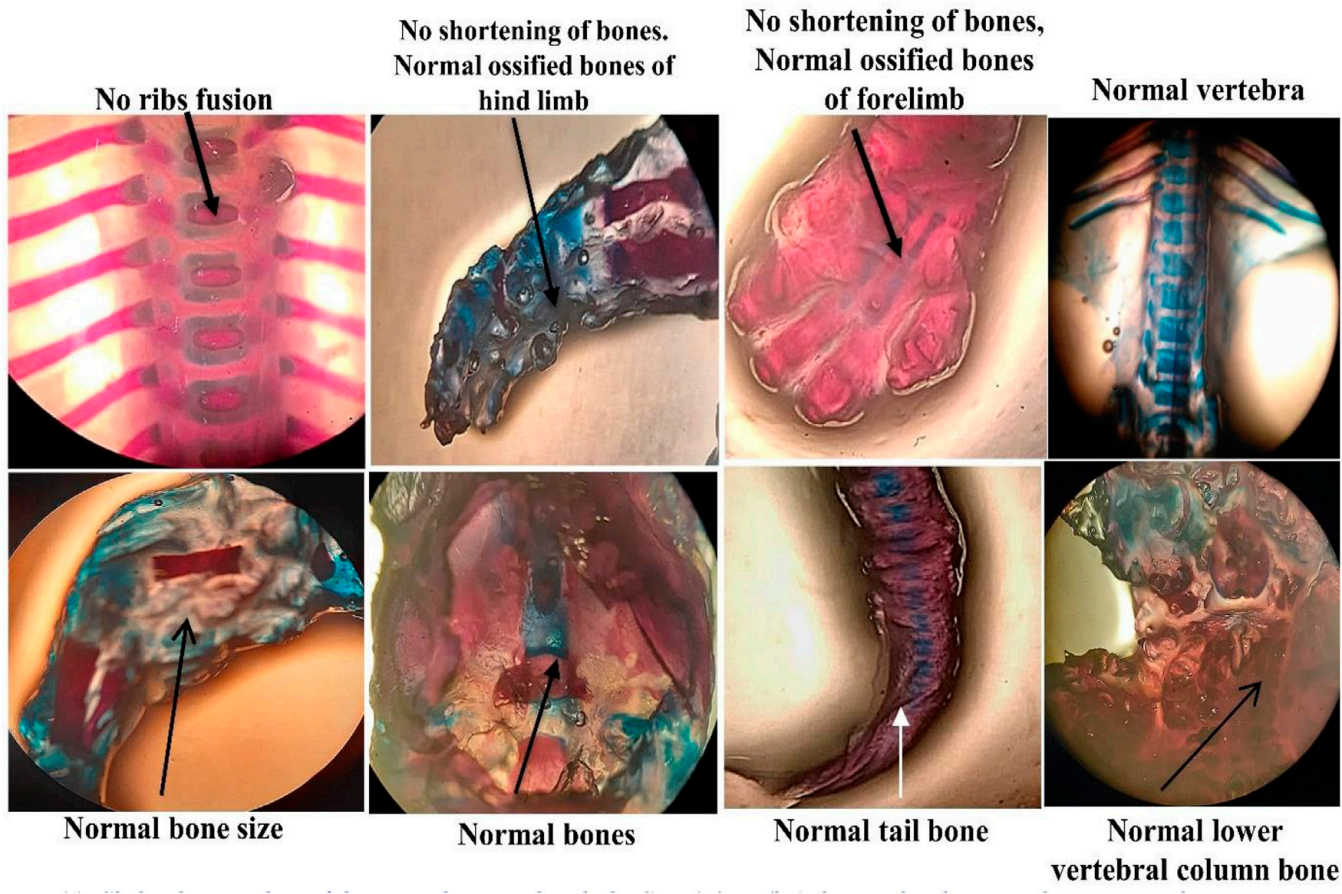
Skeletal anomalies of the animals treated with SF5 (40 mg/kg) during developmental toxicity study.

After the C-section, fetuses and placenta were carefully removed and weighed. No physical and morphological parameter was atypical by the SF5 treatment at 40 mg/kg. Hydrocephalus was observed in the fetuses after Bouin fixation. No skeletal abnormalities were observed in the teratogenic study (Figure 10, Figure 11, Table 7).

**TABLE 7:** Effect of SF5 treatment (40 mg/kg) on morphological changes in developmental toxicity.

Anomaly	Group	
	Normal control	Treated (40 mg/kg)
Cleft palate	0.0 ± 0.0	0.0 ± 0.0
Spina bifida (microns)	40 ± 0.3	42 ± 0.5
Rib malformation	0.0 ± 0.0	0.0 ± 0.0
Delayed cervical ossification	0.0 ± 0.0	0.0 ± 0.0
Early resorptions	0.0 ± 0.0	0.0 ± 0.0
Late resorptions	0.0 ± 0.0	0.0 ± 0.0
Abortions	0.0 ± 0.0	0.0 ± 0.0
No. of litters	09 ± 0.3	08 ± 0.4
No. of live fetuses	08 ± 0.3	08 ± 0.4
Maternal death rate	01 ± 0.1	00 ± 0.2
Fetal weight (gm)	5.3 ± 0.1	3.2 ± 1.3
Placental weight (gm)	0.7 ± 0.2	0.7 ± 0.2

Data are represented as mean ± SEM (*n* = 10).

#### 3.3.3 Sperm Count, Sperm Morphology, and Testosterone Level

**FIGURE 12:**
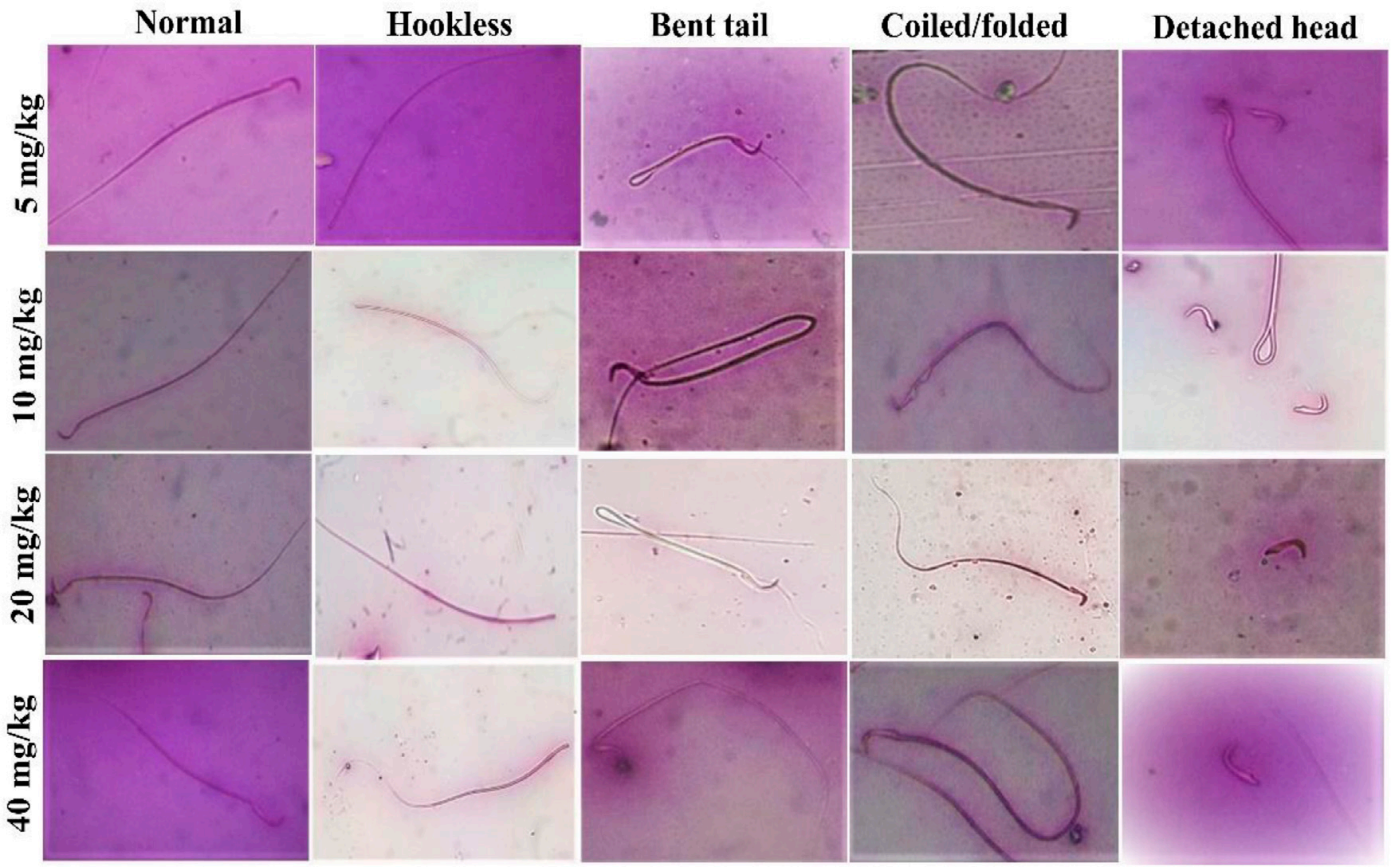
Pictorial view of morphological parameters of sperm.

**FIGURE 13:**
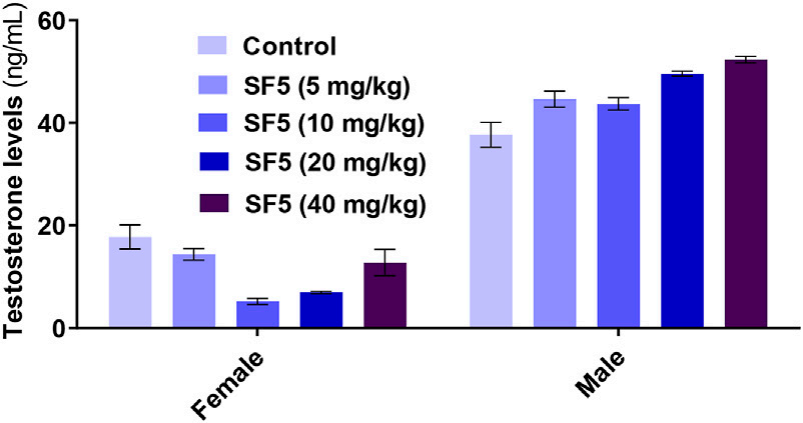
Effect of repeated-dose administration of a different dose of SF5 on testosterone levels in the serum of the treated animals in the subacute toxicity study. Data are presented as mean ± SEM, *n* = 3, ***p < 0.01 compared to the control.

The effects of SF5 on sperm morphology at selected dose levels are presented in Figure 12 and Table 8 and Figure 13. When compared with that of the control, an increased sperm count was observed at 5, 10, and 20 mg/kg with the highest count at 10 mg/kg. The dose of 40 mg/kg was the only one with lowered sperm count compared to that of the control. Similarly, the count of hookless sperms was increased at 5, 10, and 20 mg/kg and decreased at 40 mg/kg compared to that of the control. The count of normal sperms was considerably increased (3–4 times) in SF5-treated rats compared to that of the control.

**TABLE 8:** Effect of SF5 treatment at different dose levels on sperm count and its morphology.

Treatment group	Sperm count (×10^6^/ml)	Normal sperm	Hookless	Bent	Coiled/folded	Detached head
Control	157 ± 3.4	74 ± 1.2	9 ± 2.1	55 ± 3.2	8.6 ± 2.3	10 ± 0.6
SF5 (5 mg/kg)	134 ± 5.1	301 ± 0.5***	26 ± 2.3	54 ± 1.9	43 ± 1.3	10 ± 1.1
SF5 (10 mg/kg)	334 ± 5.1***	244 ± 3.1***	11 ± 2.1	60 ± 2.1	43 ± 1.1	11 ± 1.0
SF5 (20 mg/kg)	178 ± 1.6	242 ± 1.6***	36 ± 1.2	31 ± 3.2	22 ± 1.0	19 ± 2.1
SF5 (40 mg/kg)	100 ± 2.2	222 ± 1.2***	2 ± 1.0	61 ± 2.3	36 ± 1.0	1 ± 0.9

Data are presented as mean ± SEM, *n* = 5. *p < 0.05 and ***p < 0.001 are given when compared with the control.

#### 3.3.4 Histopathology

**FIGURE 14:**
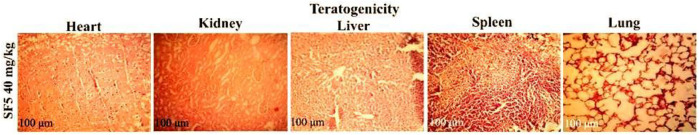
Histopathology of the selected organs in the developmental toxicity study (40 mg/kg).

At the end of developmental toxicity, different organs were isolated for any toxic effects of the SF5 on histology (Figure 14). No cellular damage was observed in the selected organs, and normal histology was seen in treated animal organs in the teratogenic study.

## 4 Discussion

Many naphthalene-based drugs are marketed to treat various diseases, such as rifampicin, naproxen, nafcillin, and bedaquiline (Anwar et al., 2021). After the detailed literature search, it was noted that pharmacological potential of SF5 is only reported in our previous study in which it was analyzed against different enzymes responsible for various diseases, such as AChE and lipoxygenase.

Preclinical toxicological studies are mandatory for establishing any molecule/compound for pharmacological and therapeutic purposes as per the standard guidelines (Sayyad et al., 2017). The major objective of the toxicity studies is to collect the results on different systems of the animals to assess the potential risks of the chemicals at high as well as ambient exposure levels. Traditionally, high-dose exposure was the main objective of the toxicity studies to find out the *LD*_*50*_ levels (Bhattacharya et al., 2011). But in a new era, it transformed it into molecular levels to determine the mechanism of action and target identification of the toxicity. The U.S. NRC in 2007 introduced a new vision and strategy in toxicity studies by evaluating the toxicity pathways. These pathways are normal cellular pathways but can be perturbed by exposure to chemicals. These new approaches are based on exploring *in vitro* assays of various key parameters of toxicity pathways, such as Nrf2 and NF-κB (Krewski et al., 2010). These toxicity pathway indicators provide valuable information regarding the risks of a substance to human health (Council, N.R., 2007; Kavlock et al., 2009).

Toxicological studies are also necessary to establish dose determination of the drugs at preclinical drug development levels (Mohs and Greig, 2017). The success rate of the drugs developed for the central nervous system (CNS) is meager, and a longer time is required for these drugs because of their safety, efficacy problems, and expensive investment (Kaitin and Milne, 2011). For the development of CNS drugs, the average time was 8–9 years that is 50% longer than that of the other drugs. Expensive investment is one of the failure cases of CNS drugs, particularly at phase 3 of clinical trials (Kaitin, 2015). The prime goal of these preclinical toxicological studies of SF5, a candidate molecule for Alzheimer’s disease (AD), is to provide sufficient evidence of the biological safety of the drugs that possess drug-like properties. Easy and cost-effective synthesis of this SF5 drug molecule makes it more suitable as a drug-adhering property molecule (Anwar et al., 2020).

An acute oral toxicity study at 2,000 mg/kg is conducted when the researcher thought that the test material is non-toxic (Prabu et al., 2013). The acute oral toxicity study at 2,000 mg/kg dose, also called the limit test, is used to identify the *LD*_*50*_ of the test compound. SF5 at 2,000 mg/kg dose did not show any mortality and morbidity, indicating that it is fairly non-toxic. However, biochemical markers were disturbed. Toxicity profiling of the test compound on long-term exposure provides valuable information regarding the cumulative toxicity of the test substances and their metabolites on the physiological and biochemical levels. The effects revealed on prolonged exposure might not be shown on acute exposure (Upadhyay et al., 2019). The subacute toxicity study was assessed using a repeated dose at 5, 10, 20, and 40 mg/kg for 28 days. No mortality or morbidity was observed throughout the study period. Animals were showed well response against the physical stimulus throughout the study. No physiological and gross behavioral changes were observed in comparison to control. Any pathological or physiological disturbance can be assessed by observing the change in the body weight because changes in the body weight are a good indicator of adverse effects generated by the test substances and their metabolites (Dybing et al., 2002). No significant change in the body weights and different organ weights was observed in the subacute toxicity study of SF5. However, at the acute toxicity study (2,000 mg/kg), the weight of the liver, lungs, and brain was increased.

Any alteration in the hematological parameters (WBCs, RBCs, platelet, and differential leukocyte count) could be presented due to the toxicity generated by the test substances (Qinna and Ghanim, 2019). SF5 at 10 mg/kg dose significantly enhanced the levels of WBCs and their differential count in females, while in males, this count was increased at all doses. This indicates the immunostimulatory behavior generated by SF5. A significant reduction in platelets was observed in the low doses of SF5 in female rats, while at high dose (40 mg/kg), the count was within the normal range compared to that of the control. The liver and kidney are the two major organs and important for toxicological studies as metabolism occurs in the liver and excretion of waste takes place in the kidney. AST and ALT are the sensitive markers evaluating the functioning of the liver. An increased level of AST indicating the hepatic cell damage and elevated levels of ALT attribute the hypertrophy (Center, 2007).

In the acute oral toxicity study, a significant rise was observed in AST and ALT levels indicating the toxic effects of SF5 on the liver. In contrast, in the subacute toxicity study, no variations were observed in AST and ALT levels in both male and female rats. AST is present in various tissues, such as the heart, kidney, liver, and skeletal muscles. AST is released due to cellular damage of any organ, while ALT is the only marker sensitive to liver performance (Ozer et al., 2008). ALP is an important marker of hepatobiliary functioning. The ALP levels are increased in cirrhosis and liver disease with unknown etiology (Ramaiah, 2007). In both acute and subacute toxicity studies, elevated ALP levels were observed, indicating the side effects of the SF5 on the liver. No effect was seen on the lipid profile of both male and female rats treated with SF5. Renal functioning was also not disturbed; however, urea levels were increased in male rats only.

There are many stress pathways present in the body that tightly regulate the different types of cell stress. Nrf2 is very important in toxicity because of its primary role in the stress response pathway. Nrf2 activation is necessary for the regulation, synthesis, and detoxification of glutathione (Jennings, 2013). In this study, no statistical difference was observed in the Nrf2 levels of normal and treated animals, indicating the absence of stress observed at tested dose levels of SF5. Reactive molecules containing oxygen are called reactive oxygen species (ROS), and these ROS molecules generated the oxidative stress pathways during toxicity. ROS play a role in normal cell signaling and homeostasis. However, excessive ROS were caused by toxicants, diseases, and radiation exposure (Deavall et al., 2012). Normal cells had a variety of defense mechanisms that prevent the cells from free radicals. These defense mechanisms contain multiple antioxidant enzymes such as SOD, CAT, and GSH (Guzik et al., 2002). SOD and CAT levels were decreased in the liver, kidney, and spleen tissues of the animals treated with a dose of 20 or 40 mg/kg SF5. Superoxide dismutase converts the free radicals into hydrogen peroxide that is further converted into water by peroxidase. Lipid oxidation provides the number of metabolites that are aldehyde and exacerbates oxidative stress. The most common polyunsaturated fatty acid peroxidation product was MDA.

MDA levels were increased in the liver, kidney, and spleen of animals treated with the high dose of SF5 in male and female rats in the subacute toxicity study. The almost same pattern was seen during the acute toxicity study. Infertility in males and females may also be produced due to the direct or indirect exposure of the drugs or chemicals. Previous studies showed that drugs enter the feta circulation by passive diffusion (Hashem et al., 2019). The diffusion of the drugs was facilitated by their low molecular weight and lipid solubility (Syme et al., 2004; Briggs et al., 2012). Our drug had low molecular weight and was non-polar. Because of this, the teratogenic study was performed using the highest dose employed in subacute toxicity studies.

Nuclear factor-kappa B (NF-κB) regulated many cellular pathways (immune response, embryogenesis, organ development, cell proliferation, apoptosis, and stress response) that control the expression of various genes that deal with the cellular or organizational stress response (Lingappan, 2018). No effect was observed in the levels of NF-κB in SF5-treated animals. No teratogenic effect was analyzed during the teratogenic study. However, animals treated with a dose level of 40 mg/kg SF5 showed hydrocephalus in pups. Only SF5 at 10 mg/kg increased the sperm count; however, all doses of the SF5 significantly increased the count of the normal sperms compared to that of the control. No effect was observed on levels of testosterone at any dose levels. Any adverse effect on cellular levels was analyzed by histopathological observations. Histological analysis showed the normal architecture of the cells in subacute and teratogenic studies.

## 5 Conclusion

The OECD and NRL guidelines recommendation provides the initial screening of chemical compounds in a scientifically acceptable way, i.e., acute, subacute, developmental toxicity, subchronic, and chronic toxicity performance and on the basis of the different toxicity pathways, respectively. This study focused on both OECD as well as NRL guidelines. This study concluded that *LD*_*50*_ of SF5 is higher than 2,000 mg/kg as no mortality was observed at this dose. Repeated dose administration did not show marked toxic effects. Hematological changes were observed in the female rats and in some biomarkers of the liver functioning test (ALP and AST), but these changes did not produce any remarkable change at the cellular and molecular levels. There are no oxidative stress pathway and cellular and adverse effects pathway initiations observed in the subacute toxicity study on both male and female rats. In addition, developmental toxicity studies revealed that SF5 did not have any teratogenic effects on pups. Toxicity profiling showed that SF5 could be a safe drug and can be used for other pharmacological purposes. However, genetoxicity, carcinogenicity, and other possible molecular mechanisms must be explored in the future studies.

## Data Availability

The raw data supporting the conclusion of this article will be made available by the authors, without undue reservation.
